# Mycotoxin tolerance affects larval competitive ability in *Drosophila recens* (Diptera: Drosophilidae)

**DOI:** 10.1093/jisesa/iead048

**Published:** 2023-06-20

**Authors:** Prajakta P Kokate, Thomas Werner

**Affiliations:** Department of Biological Sciences, Michigan Technological University, Houghton, MI, USA; Department of Biological Sciences, Michigan Technological University, Houghton, MI, USA

**Keywords:** mycotoxin tolerance, Drosophila, Drosophila recens, fitness cost, competition

## Abstract

Certain mycophagous *Drosophila* species are the only known eukaryotes that can tolerate some highly potent mycotoxins. This association between mycophagy and mycotoxin tolerance is well established because *Drosophila* species that switch hosts from mushrooms to other food sources lose their mycotoxin tolerance trait without any evolutionary lag. These findings suggest that mycotoxin tolerance may be a costly trait to maintain. In this study, we attempted to identify whether mycotoxin tolerance has a fitness cost. Larval competitive ability is a vital fitness trait, especially in holometabolous insects, where the larvae cannot move to a new host. Furthermore, larval competitive ability is known to be associated with many critical life-history traits. Here we studied whether mycotoxin tolerance adversely affects larval competitive ability on isofemale lines from 2 distinct locations. We observed that the extent of mycotoxin tolerance affected larval competitive ability, but only in isofemale lines from one location. Additionally, we observed that the high mycotoxin-tolerant isofemale lines from the same location showed poor survival to eclosion. This study shows that mycotoxin tolerance is associated with fitness costs and provides preliminary evidence of an association between local adaptation and mycotoxin tolerance.

## Introduction

Certain mycophagous *Drosophila* species can tolerate high levels of mycotoxins, including the most lethal mycotoxin, α-amanitin (Scott Chialvo and Werner 2018). This biochemical adaptation is interesting for many reasons. First, the toxic mushroom species are fewer in number (approximately 100) and therefore potentially comprise a small portion of the insect diet (Tran 2021). In other words, there appears to be a relatively small advantage in maintaining this tolerance. Second, it has been suggested that mycotoxin tolerance is a costly biochemical trait (Spicer and Jaenike 1996). In 1996, Spicer and Jaenike studied the phylogenetic relationship between 4 mycophagous and 3 nonmycophagous species within the *quinaria* group by sequencing regions of the mitochondrial cytochrome oxidase I, II, and III subunits. The most parsimonious tree constructed was used to establish a correlation between the α-amanitin tolerance and mushroom-feeding behavior. This study suggested that when a species shifts from mycophagy to another breeding site, α-amanitin tolerance is lost rapidly without any evolutionary lag (Spicer and Jaenike 1996). Thus, it is rather intriguing that the mycophagous *Drosophila* species maintain this apparently costly trait. In search of the fitness costs, in this study, we venture to identify whether mycotoxin tolerance negatively affects one particular fitness-related trait: larval competitive ability. Larval crowding is an important environmental stress, particularly in holometabolous insects, where the larvae are unable to move to a new host and must complete their development on the host where their mother laid her eggs (Kapila et al. 2021). Ovipositing females invest a large amount of energy in selecting oviposition sites to increase the chances of offspring survival (Pawlitz and Bultman 2000), where one crucial criterion for oviposition choice is to minimize competition (Diamantidis et al. 2020). Larval crowding causes scarcity of food and toxic waste accumulation, both of which can affect critical life-history traits, including development time, survival, body size, reproduction, longevity, and immune response, to name a few (Lushchak et al. 2019, Diamantidis et al. 2020, Kapila et al. 2021). Larval crowding is extensively studied in various organisms, including the model organism *Drosophila melanogaster* (Shenoi et al. 2016, Klepsatel et al. 2018, Lushchak et al. 2019). High larval densities have been shown to increase development time and longevity while reducing body size in *D. melanogaster* (Lushchak et al. 2019). *Drosophila melanogaster* that adapted to survive at high larval densities showed a better immune response against certain bacterial infections (Kapila et al. 2021). Mery et al. (2003) showed that learning ability in *D*. *melanogaster* has a constitutive fitness cost expressed as a poorer larval competitive ability. In other words, various life-history traits are affected by larval crowding, and therefore we were interested in identifying whether there is an association between mycotoxin tolerance and larval competitive ability.

Mushrooms are an ephemeral host for feeding and breeding, and extensive intraspecific and interspecific larval competition has been reported in mycophagous *Drosophila* species (Grimaldi and Jaenike 1984, Jaenike and James 1991). Food shortage and its effect on survival due to larval crowding have been reported previously (Grimaldi and Jaenike 1984). Therefore, we attempt to ask: Does mycotoxin tolerance have a fitness cost adversely affecting larval competitive ability?

## Materials and Methods

### Fly Isofemale Lines

A total of 8 *D*. *recens* isofemale lines were used for this study. These isofemale lines were selected based on a previous study (Kokate et al., 2022). Two lines with high mycotoxin tolerance and 2 lines with low mycotoxin tolerance were selected from 2 separate geographic locations: the Great Smoky Mountain National Park near Gatlinburg, TN (hereafter referred to as GSM) and *Little Bay de Noc* in Escanaba in the Upper Peninsula of Michigan (hereafter referred to as ESC). The isofemale lines were maintained on a diet of Carolina Biological Formula 4-24 Instant Drosophila medium supplemented with finely ground, freeze-dried *Agaricus bisporus* mushrooms (Oregon mushrooms, OR) in a ratio of 33.28:1, and a dental roll was added to the food vial as a pupation site. A food vial typically contained 13 ml of food. All isofemale lines were maintained at a low density (20–25 flies per food vial) to reduce variation due to rearing conditions. The flies used for the experiments were age-synchronized by collecting flies eclosed within 1–2 days to minimize variation due to parental age. The standard conditions for maintenance and experiments were 22 °C temperature under a 14 h:10 h (L:D) photoperiod at 60% humidity.

### Competition Assays

#### Experiment design

The dry food was prepared by mixing 28.3-g freeze-dried *A*. *bisporus* mushrooms (Oregon mushrooms, Oregon) and 941.9 g Carolina 4-24 Instant Drosophila medium and grinding them together into a fine powder. The competition assay vials constituted clean glass vials containing 250 mg of this finely ground powder and 1 ml of sterile distilled water.

The experimental design is depicted in Supplementary Figs. S1 and S2. Four competition assays per location were conducted. We did not perform any competition assays between isofemale lines from different locations, as the *D*. *recens* isofemale lines from GSM generally showed poorer survival than the isofemale lines from ESC.

Pilot studies were conducted to identify the number of first-instar larvae that would create low, moderate, and high larval densities. Based on the pilot study results, 8, 12, and 16 first-instar larvae, each from a low mycotoxin-tolerant line and a high mycotoxin-tolerant line, were added to create low, moderate, and high larval density vials. As shown in Supplementary Fig. S3, one replicate consisted of 3 vials, and we performed the competition assays in 5 replicates per experiment.

#### Development time, thorax length measurements, and survival

The vials were checked daily to record the time to pupation, survival to pupation, time to eclosion, and survival to eclosion. The eclosed flies were collected within 24 h by light CO_2_ anesthesia. Each fly was sexed and placed laterally under light CO_2_ anesthesia to measure the thorax length. The length from the thorax’s anterior margin to the scutellum’s posterior tip was measured and recorded as the thorax length. The thorax length of the eclosed flies was measured to the nearest 0.025 mm with an Olympus SZX16 dissection microscope fitted with an Olympus DP72 camera, using the ImageScan software (Hasson et al. 1992). The flies were then individually placed in sterile 1.5-ml Eppendorf tubes containing 500 µl of absolute ethanol. The flies were stored at −20 °C until DNA extraction could be performed.

#### Microsatellite fragment analysis

##### Identification of informative microsatellite markers

DNA was extracted from 5 flies for each isofemale line as described in Lahiri and Nurnberger (1991) and resuspended in 50 µl of Tris–EDTA buffer (pH 8.0). We used the economic 3-primer approach to identify the informative microsatellite markers and genotype the isofemale lines (Schuelke 2000). We chose the autosomal microsatellite markers previously described in Dyer et al. (2018) and modified the forward primers to have the M13(–19) tag at their 5ʹ positions. The primers used for microsatellite fragment analysis are described in Supplemental Table S1.

PCRs were performed in 25-µl reactions containing 0.05 µM of unlabeled M13-tagged forward primer, 0.2 µM of 6-carboxyfluorescein-labeled M13 primer, 0.2 µM of the reverse primer, 0.5 µl of template DNA, and 12.5 µl of 2× Amplitaq Gold polymerase.

Thermocycling conditions were 95 °C for 10 min, 92 °C (30 s), 58 °C (30 s), 72 °C (30 s) X 8 cycles, followed by 95 °C (30 s), 54 °C (30 s), 72 °C (30 s) X 25 cycles. No-template controls were included each time to rule out contamination and nonspecific PCR products. The PCR products were visualized on a 2% agarose gel.

For the microsatellite analysis, 1 µl of the PCR product, 20 µl of Hi-Di formamide (ThermoFisher, Carlsbad, CA), and 0.2 µl of GeneScan 500 LIZ dye (Thermofisher, Carlsbad, CA) were added per well of Fisherbrand 96-well PCR plate and run on an ABI 3730 Genetic Analyzer at Cornell University, Department of Biotechnology, Ithaca, NY. The analysis was performed on the Gene Scanner software version 3.0.1.

To confirm the reliability of the genotyping method, DNA was extracted individually from fresh flies of each isofemale line, and the flies were genotyped in a single-blinded study. Each isofemale line could be correctly identified using the microsatellite markers chosen.

##### Microsatellite genotyping

DNA extraction from individual flies reared from the competition assays, PCR, and the thermocycling conditions for microsatellite genotyping was performed as described in the previous section, except that the DNA was dissolved in 20 µl of Tris–EDTA buffer (pH 8.0). The marker(s) for each experiment were chosen, and each fly was genotyped based on Supplementary Table S2. A representation of how microsatellite fragment analysis was used for genotyping experimental flies is shown in Supplementary Fig. S4.

### Statistical Analyses

All statistical analyses were carried out using R version 3.6.1 (https://www.r-project.org/foundation/) and R Studio version 2021.09.2 + 382 (https://www.rstudio.com/). We used the linear mixed model (LMM) and the generalized linear mixed effects model (GLMM), implemented in R package “lme4” (Bates et al., 2015), to determine the independent variables that can explain the variation in survival, development time, and thorax lengths. We modeled fly survival using a binomial LMM with the logistic link function. We used the LMM to model development time and thorax lengths. The development time and thorax lengths were analyzed for each sex separately. To check the sufficiency of the model, the scatter plots of the deviance residuals against the predicted values were generated, and the dispersion parameter was estimated based on the ratio of the sum of squared deviance residuals and the degrees of freedom of the model if the binomial LMM was used.

To estimate whether the location of the founder parents of the isofemale lines and the extent of mycotoxin tolerance affects larval competitive ability, we first fitted a GLMM for survival to eclosion and LMM for development time and thorax lengths that includes the main effects: location (GSM or ESC), the extent of mycotoxin tolerance (high or low) and larval density (low, moderate, or high), and their 2- and 3-way interactions as the fixed effects. We then dropped different main effects starting with the 3-way interaction to identify the most parsimonious model that best explains the variance in the data. The likelihood test was used to test the significance of the models. All models included the experiment names, the isofemale lines, and the replicate vials as random effects.

## Results

### Effect of Location of the Founder Parents, the Extent of Mycotoxin Tolerance, and Larval Densities on Survival to Eclosion

The larval competitive stress imposed in our experiments was mild. Although the pilot experiment (performed on 4 isofemale lines individually) showed poor survival to eclosion on high larval density (32 larvae/food vial), when the experiments were set up using 2 isofemale lines in direct competition, the survival to eclosion among the 3 larval densities was not significantly different.

**Fig. 1.**
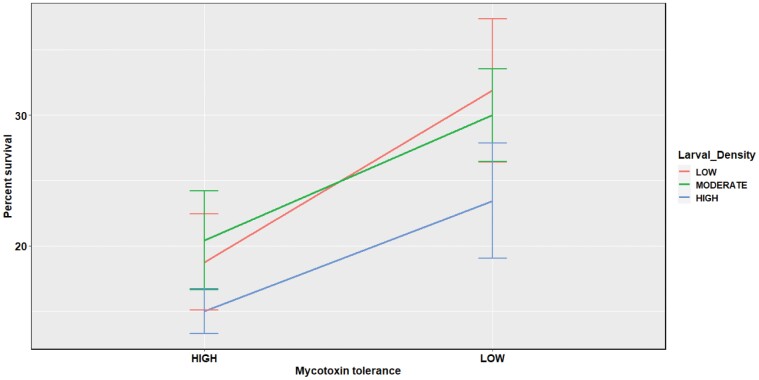
Effect of extent of mycotoxin tolerance on survival to eclosion in isofemale lines from GSM.

**Fig. 2.**
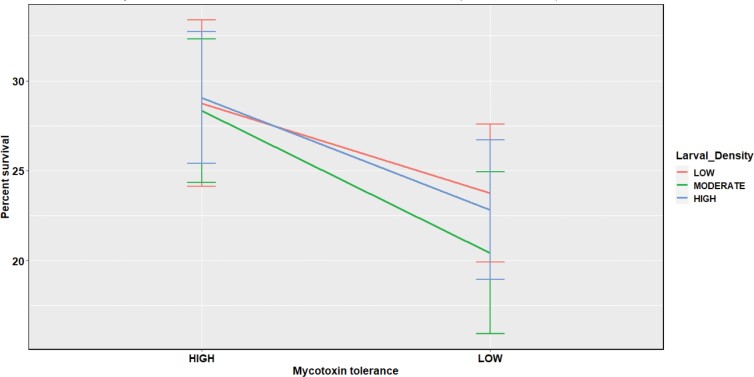
Effect of extent of mycotoxin tolerance on survival to eclosion in isofemale lines from ESC.

The 3-way interaction among the location of the founder parents, extent of mycotoxin tolerance, and larval densities was not significant. However, 2-way interaction between location and extent of mycotoxin tolerance significantly affected survival to eclosion. As shown in Figs. 1 and 2 and Tables 1 and 2, the high mycotoxin-tolerant *D*. *recens* from GSM showed poorer survival than their low mycotoxin-tolerant counterparts from GSM. On the contrary, the opposite results were found for the isofemale lines from ESC, where low mycotoxin-tolerant lines showed reduced survival.

**Table 1.** GLMM fitted to survival to eclosion data showing significant interaction between the geographical location of the founding parents and extent of mycotoxin tolerance in isofemale lines of *Drosophila recens*

Effect	LR χ^2^	DF	*P*-value
Intercept	48.622	1	0.000
Mycotoxin tolerance	4.422	1	0.035
Location	9.668	1	0.002
Mycotoxin tolerance × location	14.252	1	0.001

**Table 2.** Average percent survival to eclosion in high mycotoxin-tolerant and low mycotoxin-tolerant *Drosophila recens* isofemale lines

Location	Mycotoxin tolerance	Average percent survival ± 1 standard error		
		Larval density		
		Low	Medium	High
ESC	Low	23.8 ± 3.84	20.4 ± 4.5	22.8 ± 3.88
	High	28.8 ± 4.63	28.3 ± 3.98	29.1 ± 3.66
GSM	Low	31.9 ± 5.48	30 ± 3.55	23.4 ± 4.42
	High	18.8 ± 3.68	20.4 ± 3.8	15 ± 1.72

### Effect of Location of the Founder Parents, the Extent of Mycotoxin Tolerance, and Larval Densities on Development Time

The LMM fitted on development time data from the surviving females showed a significant 3-way interaction between the location of the founder parents, the extent of mycotoxin tolerance, and larval densities (*P*-value < 0.05, Table 3). The development time of surviving females from ESC isofemale lines did not show much difference across larval densities and the extent of mycotoxin tolerance. On the other hand, females from the high mycotoxin-tolerant isofemale lines from GSM showed a developmental delay of almost a day at high larval densities (Table 4). Although males from the high mycotoxin-tolerant isofemale lines from GSM showed a similar developmental delay (Table 5), the interaction was not statistically significant. The authors note here that female survivorship (*n* = 276) was higher than male survivorship (*n* = 231) in these assays, which may have been attributed to insignificant results in development time in males.

**Table 3.** LMM fitted to development time data in females from *Drosophila recens* isofemale lines showing significant 3-way interaction among extent of mycotoxin tolerance, larval density, and location of the founding parents on development time

	χ^2^	DF	Pr(>χ^2^)
(Intercept)	4,112.879	1	0
Mycotoxin tolerance	2.820	1	0.093
Larval density	0.241	2	0.887
Location	42.856	1	0.000
Mycotoxin tolerance × larval density	3.405	2	0.182
Mycotoxin tolerance × location	10.174	1	0.001
Larval density × location	8.960	2	0.011
Mycotoxin tolerance × larval density × location	7.896	2	0.019

**Table 4.** Development time in days in high and low mycotoxin tolerance in females from *Drosophila recens* isofemale lines at different larval densities

Location	Mycotoxin tolerance	Development time in days, mean ± 1 standard error (*n*)		
		Larval density		
		Low	Medium	High
ESC	Low	13.8 ± 0.291 (10)	13.1 ± 0.178 (13)	13.5 ± 0.167 (28)
	High	13.1 ± 0.101 (31)	13.2 ± 0.151 (33)	13.2 ± 0.092 (45)
GSM	Low	14.4 ± 0.308 (14)	14.4 ± 0.181 (25)	14.3 ± 0.167 (31)
	High	14.1 ± 0.479 (8)	14.1 ± 0.232 (21)	15.2 ± 0.327 (17)

**Table 5.** Development time in days in high and low mycotoxin tolerance in males from *Drosophila recens* isofemale lines at different larval densities

Location	Mycotoxin tolerance	Development time in days, mean ± 1 standard error (*n*)		
		Larval density		
		Low	Medium	High
ESC	Low	13.4 ± 0.217 (18)	13.6 ± 0.258 (17)	13.9 ± 0.162 (32)
	High	13.5 ± 0.247 (11)	13.8 ± 0.144 (16)	13.5 ± 0.12 (28)
GSM	Low	14.3 ± 0.172 (19)	14.5 ± 0.167 (28)	14.6 ± 0.274 (28)
	High	14.4 ± 0.371 (10)	14.5 ± 0.247 (11)	15.2 ± 0.390 (13)

### 3.3 Effect of Location of the Founder Parents, the Extent of Mycotoxin Tolerance, and Larval Densities on Thorax Length

None of the main effects—location of founding parents of isofemale lines, the extent of mycotoxin tolerance, larval density, or their interactions—had any effect on thorax lengths of the surviving males and females of the competition assays. Probably body size represented by the thorax length is a trait least perturbed by these factors.

## Discussion

The mycotoxin tolerance trait is hypothesized to have fitness costs as the trait is lost without evolutionary lag when mycophagy is lost (Spicer and Jaenike 1996). In this study, we attempted to identify whether the mycotoxin tolerance trait has a fitness cost adversely affecting larval competitive ability. Larval crowding, the associated shortage of nutrient resources, which can adversely affect survival and body size, has been demonstrated in mycophagous *Drosophila* species (Grimaldi and Jaenike 1984). Hence, understanding the effect of mycotoxin tolerance on this vital fitness trait was required.

We studied *D*. *recens*, a mycotoxin-tolerant species from the *quinaria* species group, in which high mycotoxin-tolerant and low mycotoxin-tolerant lines have been identified previously (Kokate et al., 2022). We report for the first time that the extent of mycotoxin tolerance did affect larval competitive ability in females of the *D*. *recens* isofemale lines, but only from GSM. The high mycotoxin-tolerant isofemale lines showed a developmental delay of almost a day at high larval densities.

Although we did address the question we set out to answer, we obtained additional information that requires further probing. It is not surprising that the genetic architecture of the isofemale lines founded at one location could be strikingly different from the other location. It is interesting, however, that isofemale lines from ESC appear to have no fitness cost of the mycotoxin tolerance trait affecting their larval competitive ability, whereas the isofemale lines from GSM do.

The survival-to-eclosion data of the competition assays further support this finding. The statistically significant interaction between the extent of mycotoxin tolerance and the location of the founding parents indicates that the high mycotoxin-tolerant isofemale lines from GSM show poorer survival to eclosion when compared with the high mycotoxin-tolerant isofemale lines from ESC. Kokate et al. (2022) showed that mycotoxin treatment caused poorer survival in isofemale lines from GSM compared with their ESC counterparts. Our study shows poorer survival in the high mycotoxin-tolerant isofemale lines from the GSM, even in the absence of mycotoxin treatment. Given that the 2 locations (GSM and ESC) are approximately 1,400 km apart, with distinct environmental factors, we may speculate that the selective forces at each location have contributed to this geographical variation.

Geographical variation is a common phenomenon observed across the animal and plant kingdom (Sisodia and Singh 2010, French et al. 2018, Zhang et al. 2018, Theron et al. 2019). For example, Sisodia and Singh (2010) found that *D. ananassae* from higher latitudes showed better resistance to starvation. This correlation could be attributed to the increased lipid content in these fruit flies from higher latitudes. The authors reasoned that the high lipid content was due to the carbohydrate-rich diet available at higher latitudes.

Our study is limited to 8 isofemale lines from a single species, and we cannot pinpoint the biotic or abiotic factor(s) that could have contributed to the observed geographical variation, and further research is required to identify these factors as well as differences in the genetic architecture between the flies from both locations.

The authors note here that although mycophagous *Drosophila* species and their associated mushroom toxin tolerance have been known for many years (Jaenike et al. 1983, Lacy 1984, Spicer and Jaenike 1996), due to the fastidious nature of these species (Stump 2011), research in this area has been scarce. The authors advocate that the intriguing results found in this study will pave the path for advancing research in mycotoxin tolerance.

In conclusion, our study demonstrates that the mycotoxin tolerance trait has a fitness cost that adversely affects larval competitive ability, but only in one location, suggesting a geographical variation in mycotoxin tolerance.

## Supplementary Material

iead048_suppl_Supplementary_Material

## References

[CIT0002] Bates D , Mächler M, Bolker B, Walker S. Fitting linear mixed-effects models using lme4. J Stat Softw. 2015:67(1):48.

[CIT0004] Diamantidis AD , Ioannou CS, Nakas CT, Carey JR, Papadopoulos NT. Differential response to larval crowding of a long- and a short-lived medfly biotype. J Evol Biol. 2020:33(3):329–341. 10.1111/jeb.13569 31705603 PMC7733323

[CIT0005] Dyer KA , Bewick ER, White BE, Bray MJ, Humphreys DP. Fine-scale geographic patterns of gene flow and reproductive character displacement in *Drosophila subquinaria* and *Drosophila recens*. Mol Ecol. 2018:27(18):3655–3670. 10.1111/mec.14825 PMC6360132 30074656

[CIT0006] French CM , Ingram T, Bolnick DI. Geographical variation in colour of female threespine stickleback (*Gasterosteus aculeatus*). PeerJ. 2018:6:e4807. 10.7717/peerj.4807 29785354 PMC5960269

[CIT0007] Grimaldi D , Jaenike J. Competition in natural populations of mycophagous *Drosophila*. Ecology. 1984:65(4):1113–1120. 10.2307/1938319

[CIT0008] Hasson E , Fanara JJ, Rodriguez C, Vilardi JC, Reig OA, Fontdevila A. The evolutionary history of *Drosophila buzzatii*. XXIV. Second chromosome inversions have different average effects on thorax length. Heredity (Edinb). 1992:68(Pt 6):557–563. 1612928 10.1038/hdy.1992.78

[CIT0010] Jaenike J. Induction of host preference in *Drosophila melanogaster*. Oecologia. 1983:58(3):320–325. 10.1007/BF00385230 28310329

[CIT0011] Jaenike J , James AC. Aggregation and the coexistence of mycophagous *Drosophila*. J Anim Ecol. 1991:60(3):913–928. 10.2307/5421

[CIT0012] Kapila R , Kashyap M, Poddar S, Gangwal S, Prasad NGG. Evolution of pathogen-specific improved survivorship post-infection in populations of *Drosophila melanogaster* adapted to larval crowding. PLoS One. 2021:16(4):e0250055. 10.1371/journal.pone.0250055 33852596 PMC8046209

[CIT0013] Klepsatel P , Procházka E, Gáliková M. Crowding of *Drosophila* larvae affects lifespan and other life-history traits via reduced availability of dietary yeast. Exp Gerontol. 2018:110:298–308. 10.1016/j.exger.2018.06.016 29932967

[CIT0014] Kokate P , Smith M, Hall L, Zhang K, Werner T. Inter- and intraspecific variation in mycotoxin tolerance: a study of four *Drosophila* species. Ecol Evol. 2022:12(7):e9126. 35898423 10.1002/ece3.9126 PMC9309036

[CIT0015] Lacy RC. Predictability, toxicity, and trophic niche breadth in fungus-feeding Drosophilidae (Diptera). Ecol Entomol. 1984:9(1):43–54. 10.1111/j.1365-2311.1984.tb00697.x

[CIT0016] Lahiri DK , Nurnberger JI Jr. A rapid non-enzymatic method for the preparation of HMW DNA from blood for RFLP studies. Nucleic Acids Res. 1991:19(19):5444. 10.1093/nar/19.19.5444 1681511 PMC328920

[CIT0017] Lushchak OV , Karaman HS, Kozeretska IA, Koliada AK, Zabuga OG, Pisaruk AV, Koshel NM, Mechova LV, Inomistova MV, Khranovska NM, et al. Larval crowding results in hormesis-like effects on longevity in *Drosophila*: timing of eclosion as a model. Biogerontology. 2019:20(2):191–201. 10.1007/s10522-018-9786-0 30456589

[CIT0018] Mery F , Kawecki TJ. A fitness cost of learning ability in *Drosophila melanogaster*. Proc R Soc Biol Sci. 2003:270:2465–2469. 10.1098/rspb.2003.2548 PMC1691529 14667336

[CIT0019] Pawlitz RJ , Bultman TL. Host selection by a mycophagous fly and its impact on fly survival. Ecography. 2000:23(1):41–49. 10.1111/j.1600-0587.2000.tb00259.x

[CIT0020] Schuelke M. An economic method for the fluorescent labeling of PCR fragments. Nat Biotechnol. 2000:18(2):233–234. 10.1038/72708 10657137

[CIT0021] Scott Chialvo CH , Werner T. Drosophila, destroying angels, and deathcaps! Oh my! A review of mycotoxin tolerance in the genus *Drosophila*. Front Biol. 2018:13(2):91–102. 10.1007/s11515-018-1487-1

[CIT0022] Shenoi VN , Ali SZ, Prasad NG. Evolution of increased adult longevity in *Drosophila melanogaster* populations selected for adaptation to larval crowding. J Evol Biol. 2016:29:407–417. 26575793 10.1111/jeb.12795

[CIT0023] Sisodia S , Singh BN. Resistance to environmental stress in *Drosophila ananassae*: latitudinal variation and adaptation among populations. J Evol Biol. 2010:23(9):1979–1988. 10.1111/j.1420-9101.2010.02061.x 20695963

[CIT0024] Spicer GS , Jaenike J. Phylogenetic analysis of breeding site use and alpha-amanitin tolerance within the *Drosophila quinaria* species group. Evolution. 1996:50(6):2328–2337. 10.1111/j.1558-5646.1996.tb03620.x 28565683

[CIT0025] Stump AD , Jablonski SE, Bouton L, Wilder JA. Distribution and mechanism of alpha-amanitin tolerance in mycophagous *Drosophila* (Diptera: Drosophilidae). Environ Entomol. 2011:40(6):1604–1612. 10.1603/EN11136 22217779

[CIT0026] Theron GL , de Waal C, Barrett SCH, Anderson B. Geographic variation of reproductive traits and competition for pollinators in a bird-pollinated plant. Ecol Evol. 2019:9(18):10122–10134. 10.1002/ece3.5457 31673331 PMC6816071

[CIT0027] Tran HHJA. 2021. Mushroom toxicity. Treasure Island (FL): StatPearls Publishing.

[CIT0028] Zhang C , Jiang T, Lu G, Lin A, Sun K, Liu S, Feng J. Geographical variation in the echolocation calls of bent-winged bats, *Miniopterus fuliginosus*. Zoology. 2018:131:36–44. 10.1016/j.zool.2018.05.005 29803625

