# Supplementary material for: Mycotoxin tolerance affects larval competitive ability in Drosophila recens (Diptera: Drosophilidae)
Source: J Insect Sci. 2023 Jun 20;23(3):18. doi: 10.1093/jisesa/iead048 (PMC10281370; doi:10.1093/jisesa/iead048)
Supplement: iead048_suppl_Supplementary_Material [file iead048_suppl_supplementary_material.docx]

**A**


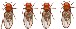

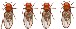

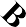

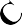


***D*. *recens* E05#53**

**(High mycotoxin-tolerant)**

***D*. *recens* E10#56**

**(Low mycotoxin-tolerant)**

**D**


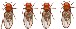

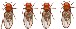


***D*. *recens* E01#49**

**(High mycotoxin-tolerant)**

***D*. *recens* E15#11**

**(Low mycotoxin-tolerant)**

**Supplemental Figure 1:** Illustration of the experimental design for isofemale lines from ESC. Each double arrow indicates one larval competition experiment. Two high mycotoxin-tolerant and two low mycotoxin-tolerant lines are being used to set up four experiments. Strain numbers consist of E (Escanaba) followed by a two-digit collection site number, followed by the two-digit isofemale line number.

**P**


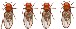

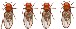

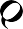

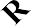


***D*. *recens* GSM17**

**(High mycotoxin-tolerant)**

***D*. *recens* GSM26**

**(Low mycotoxin-tolerant)**

**S**


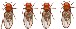

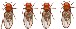


***D*. *recens* GSM5**

**(High mycotoxin-tolerant)**

***D*. *recens* GSM35**

**(Low mycotoxin-tolerant)**

**Supplemental Figure 2:** Illustration of the experimental design for isofemale lines from GSM. Each double arrow indicates one larval competition experiment. Two high mycotoxin-tolerant and two low mycotoxin-tolerant lines are being used to set up four experiments. Strain numbers consist of GSM (Great Smoky Mountains), followed by the isofemale line number.

**Low larval density Moderate larval density High larval density**

**Replicate # 1**

**8 + 8**

**12 + 12**

**16 + 16**

**Replicate # 2**

**8 + 8**

**12 + 12**

**16 + 16**

**8 + 8**

**12 + 12**

**16 + 16**

**Replicate # 3**

**Replicate # 4**

**8 + 8**

**12 + 12**

**16 + 16**

**Replicate # 5**

**8 + 8**

**12 + 12**

**16 + 16**

**Supplemental Figure 3:** Diagram showing an example of the larval competition assay. Each assay comprised five replicates, as shown in the Figure. The numbers in green represent the first-instar larvae added from the low mycotoxin-tolerant isofemale line and the numbers in red represent the first-instar larvae added from the high mycotoxin-tolerant isofemale line.

**Supplemental Table 1:** Primer information for microsatellite fragment analysis.

| **Marker/ Primer name** | **Primer sequence** | **Expected size in bp** |
| --- | --- | --- |
| Drec1007 fwd | CACGACGTTGTAAAACGACTTTCATTAGCACGCGCA AC | 158-184 |
| Drec1007 rvs | CGATTGCTTTTTGCCTTTG |  |
| Drec1008 fwd | CACGACGTTGTAAAACGACGCAGAGCCAGGCA TAAAATG | 124-220 |
| Drec1008 rvs | GGCTTCAATTTTGCCACAC |  |
| Drec1024 fwd | CACGACGTTGTAAAACGACCGGAACTGAACTG AAACTGC | 398-466 |
| Drec1024 rvs | TCATTGTCAGTGCCAAAAGTG |  |
| Drec1079 fwd | CACGACGTTGTAAAACGACGCAAATTTATTAGG TGCACAGC | 195-241 |
| Drec1079 rvs | ACGGAATGGGTGAGAAAGAG |  |
| Drec1081 fwd | CACGACGTTGTAAAACGACGCAATAAAACTAG CAAAACAAATAGC | 156-195 |
| Drec1081 rvs | GCGTGTGCAGCTTGTAAATG |  |
| Drec1083 fwd | CACGACGTTGTAAAACGACCCAATGTGTTAGTG TGTTATGAGTG | 151-230 |
| Drec1083 rvs | CCGAAGTTGCGAATATTTTG |  |
| Drec2002 fwd | CACGACGTTGTAAAACGACTTCGTCCAAGCATG AAAATG | 358-420 |
| Drec2002 rvs | GGAAACGGAAACGTGGATAC |  |
| Drec2004 fwd | CACGACGTTGTAAAACGACGACGCAAATCAAA TGTCGTG | 331-366 |
| Drec2004 rvs | CATATTTCGCTGCAGCTTTC |  |
| Drec2084 fwd | CACGACGTTGTAAAACGACTTTATGAATGAGTT GGGCACAC | 205-275 |
| Drec2084 rvs | GTCGCCTTGGGGATATTTG |  |

| Drec2085 fwd | CACGACGTTGTAAAACGACAAACACGTCTAAG CCAAGCTG | 203-253 |
| --- | --- | --- |
| Drec2085 rvs | GACGCCGCATAAATTAGCC |  |
| Drec3028 fwd | CACGACGTTGTAAAACGACGTGGCATCGTTGGC AAATC | 111-138 |
| Drec3028 rvs | AACATTGATGCCAGCAAGC |  |
| Drec3038 fwd | CACGACGTTGTAAAACGACTTTCAACTCGGCTG CTTTTC | 275-355 |
| Drec3038 rvs | CTGACTGTCCGGCTCAATG |  |
| Drec3047 fwd | CACGACGTTGTAAAACGACATGCTTGTGACGAT GCTTTG | 461-527 |
| Drec3047 rvs | TGCATTCTTGCATTCCTTTTC |  |
| Drec3089 fwd | CACGACGTTGTAAAACGACCGATGTCGAACAC ATAAAAAGTG | 387-440 |
| Drec3089 rvs | AAGGCAAATTCTATTCAACTAAATG |  |
| Drec3090 fwd | CACGACGTTGTAAAACGACCCAGCAACTGGTA ATTTGGAC | 180-226 |
| Drec3090 rvs | AGGAAAGGCAAGGCAAGAAG |  |
| Drec3091 fwd | CACGACGTTGTAAAACGACTATGTGCTCGGGCG TGTG | 143-179 |
| Drec3091 rvs | GGCGGGTACGTATTTGTTTC |  |
| Drec4069 fwd | CACGACGTTGTAAAACGACCCCCACATCGTCAT CAAAG | 197-245 |
| Drec4069 rvs | TGCACACCGATGAGTACGAG |  |
| 6-  Carboxyfluor escein- labelled M13 primer | CACGACGTTGTAAAACGAC | Not applicable |

**Supplemental Table 2:** Informative microsatellite markers.

| Experiment name | Isofemale lines used | Microsatellite marker | Expected size (bp) |
| --- | --- | --- | --- |
| A | *D*. *recens* E05#53 | Drec1008 | 111/163 |
|  | *D*. *recens* E10#56 | Drec1008 | 128/139 |
| B | *D*. *recens* E05#53 | Drec1008 | 111/163 |
|  | *D*. *recens* E15#11 | Drec1008 | 142 |
| C | *D*. *recens* E01#49 | Drec1083 | 146/156 |
|  | *D*. *recens* E10#56 | Drec1083 | 160/174 |
| D | *D*. *recens* E01#49 | Drec1083 | 146/156 |
|  | *D*. *recens* E15#11 | Drec1083 | 165 |
| D | *D*. *recens* E01#49 | Drec1024 | 413/426 |
|  | *D*. *recens* E15#11 | Drec1024 | 409/421 |
| P | *D*. *recens* GSM17 | Drec2085 | 185/211 |
|  | *D*. *recens* GSM26 | Drec2085 | 206/238 |
| Q | *D*. *recens* GSM17 | Drec3090 | 189/191 |
|  | *D*. *recens* GSM35 | Drec3090 | 174/180 |
| R | *D*. *recens* GSM5 | Drec1081 | 142/147/149/153 |
|  | *D*. *recens* GSM26 | Drec1081 | 137/157 |
| S | *D*. *recens* GSM5 | Drec1079 | 214 |
|  | *D*. *recens* GSM35 | Drec1079 | 208/210 |

| A.  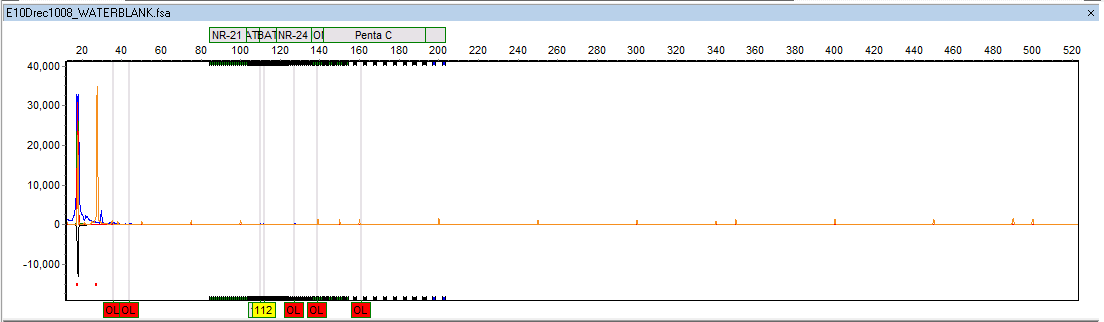 |
| --- |
| B.  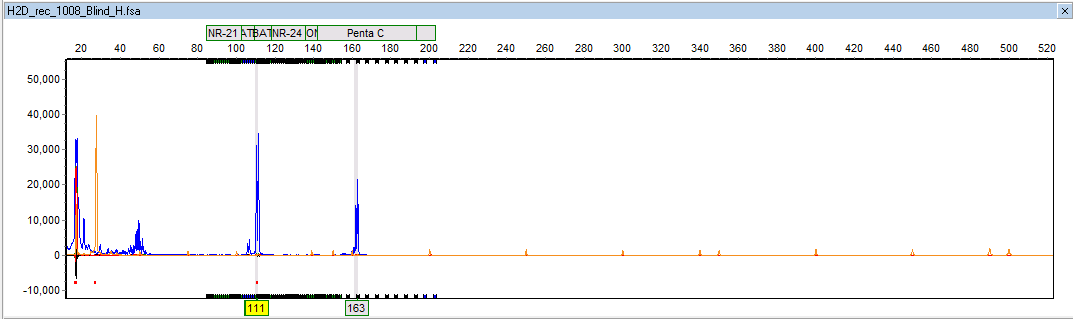 |
| C.  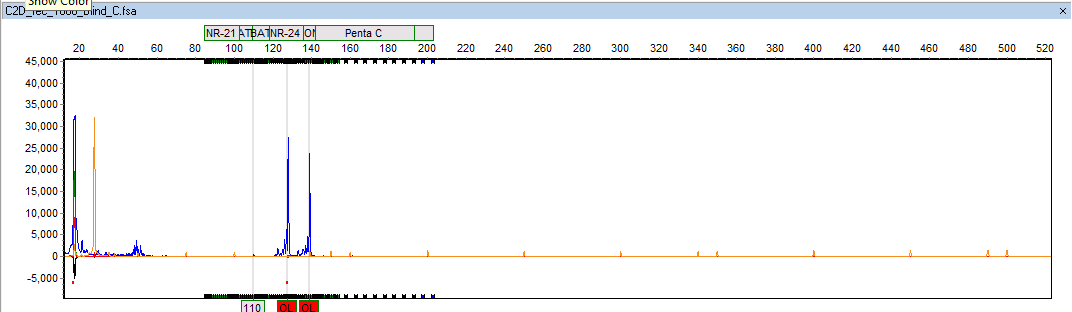 |

8

| D.  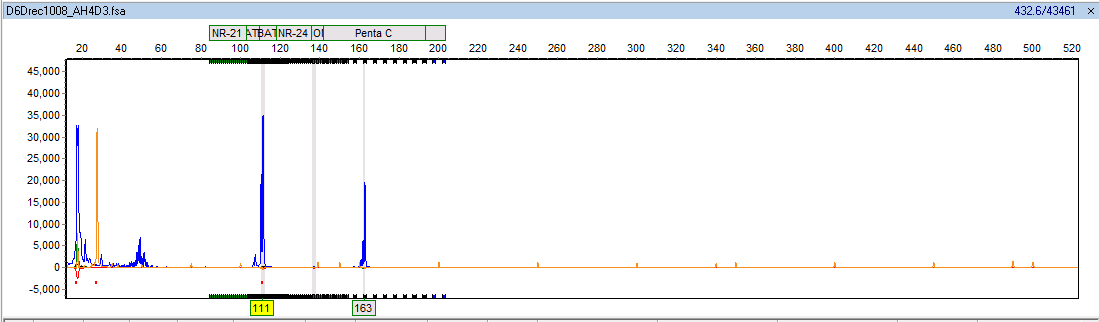 |
| --- |
| E.  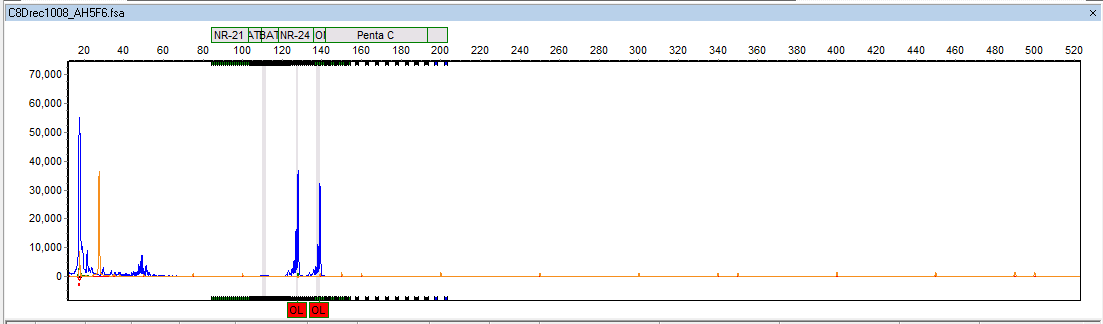 |

**Supplemental Figure 4:** Microsatellite fragment analysis example for Experiment A, using the marker Drec1008. A. Water Blank, B. Sample *D*. *recens* E05#53, C. Sample *D*. *recens* E10#56, D. Sample AH4D3 (Experiment A, High larval density, replicate #4, dead fly #3), E. Sample AH5F6 (Experiment A, High larval density, replicate #5, female fly.
